# Data-Prompted Interviews: Using Individual Ecological Data to Stimulate Narratives and Explore Meanings

**DOI:** 10.1037/hea0000234

**Published:** 2015-05-25

**Authors:** Dominika Kwasnicka, Stephan U. Dombrowski, Martin White, Falko F. Sniehotta

**Affiliations:** 1 Institute of Health and Society, Newcastle University and Fuse UKCRC Centre for Translational Research in Public Health; 2 School of Natural Sciences, Division of Psychology, University of Stirling; 3 UKCRC Centre for Diet and Activity Research (CEDAR) and MRC Epidemiology Unit, School of Clinical Medicine, University of Cambridge; 4 Institute of Health and Society, Newcastle University and Fuse UKCRC Centre for Translational Research in Public Health

**Keywords:** data-prompted interview, ecological momentary assessment, qualitative methods

## Abstract

***Objective:*** An emerging trend in qualitative research is to use individual participant data to stimulate narratives in interviews. This article describes the method of the data-prompted interview (DPI) and highlights its potential benefits and challenges. ***Method:*** DPIs use personal ecological data gathered prior to the interview to stimulate discussion during the interview. Various forms of data can be used including photographs, videos, audio recordings, graphs, and text. This data can be gathered by the researcher or generated by the participant and may utilize ecological momentary assessment. ***Results:*** Using individual data in DPIs can stimulate visual and auditory senses, enhance memory, and prompt rich narratives anchored in personal experiences. For the researcher, DPIs provide an opportunity to explore the meaning of the data and to explain data patterns. For the participant, presented stimuli give guidance for discussion and allow them to reflect. The challenges associated with conducting DPIs include practical issues such as data selection and presentation. Data analyses require narratives to be interpreted together with the data. Ethical challenges of DPI include concerns around data anonymity and sensitivity. ***Conclusions:*** Combining various sources of data to stimulate the interview provides a novel opportunity to enhance participants’ memories and to meaningfully assess and analyze data patterns. In the context of health promotion and illness prevention, DPI offers a unique opportunity to explore reasons, opinions, and motivations for health-related behaviors in the light of previously gathered data.

## Data-Prompted Interview: Definition and Aims

Data-prompted interviews (DPIs) use personalized prompts such as photos, videos, audio recordings, graphs and text to stimulate discussion in a qualitative interview setting. In line with other qualitative research methods, DPIs are primarily explanatory and are used to generate in-depth understanding of human behaviors and experiences, taking into account complexity, detail, and context (Ritchie, Lewis, Nicholls, & Ormston, 2013). Qualitative interviews can be defined as a “conversation with a purpose” (Gerard Sister, 1959). DPIs may add depth to this conversation. The three main aims of DPIs are:

1 To actively stimulate and guide the discussion using data-driven prompts
2 To explore, integrate and contrast interpretations derived from data with participant’s experiences and narratives
3 To discuss and evaluate participants’ views toward the personal data presented.

DPIs can be solely qualitative (e.g., photographs discussed in the DPI) or they can use a mixed method approach (e.g., combining quantitative measures with DPI).^1^

## Method

### Data-Prompted Interview: Sources of Data

DPIs use various sources of data to stimulate discussion during an interview. In this section three common sources of data are discussed, namely (a) photographs, (b) video or audio recordings, and (c) graphical representations of quantitative data. However, other sources can also be used, for instance material objects; for example, home possessions (Miller, 2008) and artworks (Radley & Bell, 2007).

#### Photographs

Photographs are used in interviews through photo elicitation and visual storytelling. Photo elicitation is a qualitative research technique that uses a photograph to stimulate a discussion (Harper, 2002). For example, an individual trying to lose weight could take photographs of key motivators and barriers to their everyday weight-loss attempts. In visual storytelling participants are encouraged to tell a story that emerges from the sequence of pictures (Drew, Duncan, & Sawyer, 2010). Photographs have been used in interviews to extend and elaborate on memories and evoke emotions (Clark-Ibáñez, 2004; Harper, 2002), to stimulate enriched narratives about health-related experiences; for example, living with illness (Bell, 2002) or experiencing poverty (Hodgetts, Radley, Chamberlain, & Hodgetts, 2007).

Images are processed faster than verbal cues and they evoke different brain regions (Harper, 2002). Photo elicitation covers a range of approaches; for example, asking participants to proactively take pictures documenting specific issues or experiences, discussing relevant photos already taken by participants, as well as elaborating on photos provided by the researcher. Photo elicitation facilitates data collection among harder to research groups, such as children (Drew et al., 2010) and indigenous communities (Samuels, 2004), as it can be easier for them to express themselves through photographs than through words. For some studies, photo elicitation has been found to be more appropriate than other forms of data collection. For instance, photo elicitation was more effective than daily diaries in collecting data on self-management of diabetes; participants enjoyed capturing information in pictures but they did not adhere with a daily diary (Thompson & Oelker, 2013).

#### Video or audio recordings

Video or audio recordings are employed in DPIs through video- or audio-elicitation; in video- or audio-elicitation studies, recorded visual and audio material is used to initiate and focus the discussion during the interview (Coleman, Murphy, & Cheater, 2000; Gao, Burke, Somkin, & Pasick, 2009; Saba et al., 2006). For instance, recording of a physician and patient interaction can be used as a tool to stimulate a discussion about the encounter (for a good overview of the topic see: Henry & Fetters, 2012). Video elicitation can also be used for teaching purposes (Chou & Lee, 2002) or as a health intervention tool (Harms et al., 2004). Video elicitation can be used to aid memory and to gain insights into thoughts, emotions, and beliefs regarding the recorded situation. Video-elicitation studies with children suggest that film is a powerful tool to enable young people to meaningfully engage in health research (Bissell, Manderson, & Allotey, 2000). Video diaries are particularly suited to research with people from vulnerable groups, giving them tools to express their shared viewpoints; for example, recording situations relating to shared community issues that affect health (Brown, Costley, Friend, & Varey, 2010). Video and audio elicitation aids memory and the interview gives the participant an opportunity to reflect on the recordings.

#### Graphical representations

Graphical representations including graphs, diagrams, statistics, and maps can be used as stimuli in interviews. Several forms of graphical representation can be used to evoke discussion. For instance a “life grid” is a visual tool used for mapping important life events against a time period to construct and reflect on a participant’s life story (Wilson, Cunningham-Burley, Bancroft, Backett-Milburn, & Masters, 2007). Another example is “chart-stimulated recall,” a technique that uses hospital patient charts to probe physician recall and provide context about barriers and facilitators to effective care (Guerra et al., 2007). Diagrams are effective instruments in conveying complex thoughts to others; they often show relationships and concepts that are difficult to explain verbally (Crilly, Blackwell, & Clarkson, 2006). Using graphical representations in interviews was reported to be helpful while discussing sensitive issues (Kesby, 2000; Wilson et al., 2007) and while conducting research with children (Bagnoli, 2009; Young & Barrett, 2001). Multiple health-related studies have employed graphical representations of data to stimulate discussion (e.g., Kesby, 2000; Wilson et al., 2007). Graphical representations often involve higher levels of mental processing and can provoke and facilitate discussion that might have been too complex without a visual prompt.

#### Mixed sources of data

DPIs can draw on multiple sources and types of data. A study examining disabled young men transitioning to adulthood, for instance, combined audio diaries and photography, using them in interviews suggesting that participants’ acts of gathering data (data creation) are analyzable events in themselves (Gibson et al., 2013). Another example is “timelining”, a method developed in the context of weight-management research (Sheridan, Chamberlain, & Dupuis, 2011). In this research participants’ weight over time was plotted on a graph and elaborated on by a variety of stimuli such as photographs, medical records, and personal diaries. The timeline was used as a prompt in the interview to document, record, and extend understanding of participants’ experiences, encouraging rich temporal narratives (Sheridan et al., 2011). A growing body of qualitative health research uses photos, videos, and graphics to stimulate the discussion; some studies combine more than one data source (Gibson et al., 2013; Sheridan et al., 2011).

### Data Generation

There are two main data-generation categories: researcher-created data and participant-created data (Prosser & Loxley, 2008). Data presented to the participant can be captured automatically, for example, with accelerometry or with GPS measures (Oliver, Badland, Mavoa, Duncan, & Duncan, 2010). Data can also be gathered by a participant through Ecological Momentary Assessment (EMA). EMA involves repeated assessment of an individual’s behaviors and/or experiences in real time, in their natural environment (Shiffman, Stone, & Hufford, 2008). EMA often involves prompting participants at random or prespecified times and asking them to collect the data (e.g., take a picture, make a video, answer questions). EMA can also be used in *N*-of-1 research designs where hypotheses about relationships between variables (Quinn, Johnston, & Johnston, 2013) or responses to interventions (Sniehotta, Presseau, Hobbs, & Araújo-Soares, 2012) can be tested at the individual level. Data gathered through EMA is subsequently used in the interview.

EMA can be participant-initiated or researcher-initiated. For instance participants of a smoking cessation study were asked to report any episodes of smoking as they happened and were asked to complete a brief assessment of their craving, mood and context during the episode (participant-initiated assessment). In addition, participants received a similar assessment at random times during the day (researcher-initiated assessment) (Shiffman, 2005). Data generation methods vary depending on who generates the data, how and when it is gathered and subsequently how is it used for an interview. EMA can identify levels and changes in measures within the individual (e.g., in weight or mood) or relationships between measures (e.g., relationships between pain and physical activity over time). This creates novel and highly personalized stimuli in interviews as participants might not be aware of these relationships before being presented with the EMA results.

## Results

### Benefits and Potential Challenges of DPI

There are various benefits and some potential challenges of DPIs. DPIs use prompts to trigger discussion by stimulating verbal, visual, and auditory senses. The presentation of the stimuli evokes various brain regions involved in nonverbal information processing, including specific brain regions that play a role in memory, attention, logical analysis, and processing emotions (Kandel, Schwartz, & Jessell, 2000). DPIs evoke memories; participants are presented with data that stimulates discussion and encourages reflection (Clark-Ibáñez, 2004; Harper, 2002; Henry & Fetters, 2012). DPIs provide unique opportunities to clarify and evaluate the meaning of the data and to discuss thoughts and emotions underlying the data (Sheridan et al., 2011). This refers to thoughts and feeling stimulated about the data during the interview and those represented by the data when it was generated. If the data used for DPIs is covert to the participants then their expectations regarding the data can be explored. Dependent on the research paradigm and philosophical underpinnings, exploring relationships between objective data and the narratives provided by the research participant can also be explored as means to validate the data.

There are also several advantages of DPIs for research participants. Data provides topics and direction for discussion and it serves as a point of reference for interview questions (Sheridan et al., 2011). DPIs provide an opportunity for researchers and researched to coproduce knowledge by adding the participant’s perspective to the researcher’s and agreeing to an interpretation. In the context of health-related behaviors such as smoking, drinking, or binge eating, changes in perceptions or single events of relapse can easily be forgotten. Enhancing participants’ narratives with data prompts gives health researchers a unique opportunity to gain a broader understanding of underlying health-related cognitions and contexts. Data can be used as a persuasive presentation tool. Knowledge gained through DPI can also inform future health interventions; for example, enhancing shared decision making in health settings.

There are also some potential practical, analytical, and ethical challenges in using DPIs. Practical challenges relate to the data-selection process. Data needs to be carefully selected (Harper, 2002; Henry & Fetters, 2012) and clearly explained to the participant. Analysis of DPI data can be challenging as prompts need to be stored together with the interview recording. Data needs to be analyzed and interpreted in conjunction with the interview narratives. Some ethical challenges also need to be addressed before the interview concerning data anonymity and sensitivity. Participants need to consent to gather and discuss data they are presented with. Data gathered can include personal and sensitive information regarding participants and their contexts. Other people may inherently be part of the generated data (e.g., spouses of participants), so they also need to consent to appear on the pictures or videos; lack of consent may prevent analysis and publication. Vulnerable groups have been reported as receptive and easy to collaborate with in data-prompted research (Brown et al., 2010; Drew et al., 2010). Nevertheless, special ethical challenges, such as parent/carer consent, should be addressed while working with these groups.

## Conclusions

In qualitative interviews participants refer to their beliefs, insights, and awareness at the time of the interview. Participants’ narratives are based on memories, which are often limited. Using data as stimuli for an interview can enhance memory and allow a high level of participant-led involvement in health research, as well as providing stimuli for eliciting further verbal material. DPI as a research method has a potential to enhance our understanding of health-related issues and to intervene to change health-related behaviors.

## Footnotes

1 This article focuses on one-to-one interviews. However, data-prompted discussion can also take place in focus groups (for instance: Cooper & Yarbrough, 2010), and most of the DPI characteristics described in this paper also apply to data-prompted focus groups.
